# Sublobectomy versus Lobectomy for stage IA (T1a) non-small-cell lung cancer: a meta-analysis study

**DOI:** 10.1186/1477-7819-12-138

**Published:** 2014-05-01

**Authors:** Yaxin Liu, Cheng Huang, Hongsheng Liu, Yeye Chen, Shanqing Li

**Affiliations:** 1 Department of Thoracic Surgery, Peking Union Medical College Hospital, Shuaifuyuan No.1 Dongcheng District, Beijing 100730, China; 2 Graduate School of Peking Union Medical College, Chinese Academy of Medical Sciences, Shuaifuyuan No.1 Dongcheng District, Beijing 100730, China

**Keywords:** Sublobectomy, Lobectomy, Non-Small-Cell lung Cancer, Meta-analysis, Overall Survival (OS)

## Abstract

**Background:**

Although lobectomy is considered the standard surgical treatment for the majority of patients with non-small-cell lung cancer (NSCLC), the operation project for patients with stage IA NSCLC (T1a, tumor diameter ≤2 cm) remains controversial. Sublobectomy is appropriate only in certain patients as many doctors consider it to be overtreatment. We evaluated the five-year overall survival rate of sublobectomy and lobectomy for stage IA NSCLC (T1a, tumor diameter ≤2 cm) through a meta-analysis.

**Methods:**

The five-year overall survival rate (OS) of stage IA (T1a) NSCLC after sublobectomy (including wedge resection and segmentectomy) and lobectomy were compared. We also compared the OS of stage IA (T1a) NSCLC after segmentectomy and lobectomy. The log (hazard ratio, ln (HR)) and its standard error (SE) were used as the outcome measure for data combining.

**Results:**

There were 12 eligible studies published between 1994 and 2013 in which the total number of participants was 18,720. When compared to lobectomy, there was a statistically significant difference of sublobectomy on OS of stage IA (T1a) NSCLC patients (HR 1.38; 95% confidence interval (95% CI), 1.19 to 1.61; *P* <0.0001). For the comparison between segmentectomy and lobectomy, there was also a statistically significant difference of segmentectomy alone on OS of stage IA (T1a) NSCLC patients (HR 1.48; 95% CI: 1.27 to 1.73; *P* <0.00001)

**Conclusions:**

We have concluded that in stage IA (T1a) patients sublobectomy, including segmentectomy and wedge resection, causes a lower survival rate than lobectomy.

## Background

With the wide use of high-resolution computed tomography (CT) and low-dose helical CT in lung cancer screening, an increasing number of non-small-cell lung cancers (NSCLCs) are diagnosed at the early T1 stage. Considering the relatively good prognosis of T1 stage NSCLC, many surgeons began to question the necessity of a total lobectomy in the management of patients with such small lesions [1-3]. As an alternative, sublobectomy, including wedge resection and segmentectomy, reduces the length of surgery, thus resulting in fewer complications and shorter hospitalization time [4,5]. This procedure is only acceptable in cases with a tumor size of less than 2 cm, according to the recommendation from the National Comprehensive Cancer Network (NCCN) Clinical Practice Guideline in Oncology for NSCLC, version 2.2013, however the outcome of sublobectomy is still controversial.

In 1995, the Lung Cancer Study Group (LCSG) compared the outcomes of sublobar with lobar resection in a randomized trial and demonstrated that patients with sublobar resection had a lower survival rate [6]. The same results have been published in some non-randomized trials [7], which examine a comparison between wedge resection alone and lobectomy. However, several other studies have demonstrated that limited resection was not inferior to lobectomy regarding prognosis in patients with small and peripheral NSCLC [8]. The results from the LCSG have been further challenged by several retrospective studies, which have suggested that limited resection might be equally effective in the treatment of stage IA (T1a) patients with a tumor size of less than 2 cm compared with lobectomy, particularly among elderly patients [9-12].

To compare the outcome of sublobectomy and lobectomy, Nakamura *et al.* conducted a meta-analysis of published studies between 1970 and 2004 in 2005 [10]. The analysis indicated that the outcome of limited resection was comparable to lobectomy in patients with stage I lung cancer. Since then, several large clinical trial results have been published. In 2012, Fan *et al.* conducted another meta-analysis of published studies between 1990 and 2010 which indicated that for stage I patients, sublobectomy causes a lower survival rate than lobectomy, while for stage IA (T1a) patients, sublobectomy produces a similar survival rate to lobectomy [13]. Taking all these into consideration, we felt it necessary to reevaluate the efficacy of sublobectomy in patients with stage IA (T1a) NSCLC (tumor diameter ≤2 cm). We therefore collected the updated data from published studies between 1994 and 2013 and evaluated the effectiveness of sublobectomy compared to lobectomy for stage IA (T1a) NSCLC (tumor diameter ≤2 cm) through a meta-analysis of these studies.

## Methods

### Identification of studies

We searched PubMed using the strategy of (limited resection [Title/Abstract] OR (sublobar resection [Title/Abstract]) OR (segmentectomy [Title/Abstract]) OR (wedge resection [Title/Abstract]) AND (lung [Title/Abstract] OR pulmonary [Title/Abstract]) AND (cancer [Title/Abstract]) OR (carcinoma [Title/Abstract]) AND (lobectomy [Title/Abstract]). We also searched Embase by the strategy of ‘limited resection’: ab OR ‘sublobar resection’: ab OR segmentectomy: ab OR ‘wedge resection’: ab AND (lung: ab OR pulmonary: ab) AND (cancer: ab OR carcinoma: ab) AND lobectomy: ab. The citations of all retrieved articles were checked to identify any other potentially relevant publications. The studies in the search results were selected based on the inclusion and exclusion criteria. The inclusion criteria were: (i) outcomes of interest include overall survival rate (OS) (ii) articles were peer-reviewed, published, and original articles (iii) information was described in the article on how the hazard ratio (HR) and standard error (SE) can be calculated (iv) study subjects had to be limited to clinical stage IA (T1a) patients, (v) patients were divided into lobectomy and sublobectomy, (vi) if the enrolled patients were from the same institutions and in the same period, only the most recently published data would be enrolled into the study. The exclusion criteria were: (i) letters to editor, case reports, reviews, and non-English articles (ii) patients were divided into segmentectomy and wedge resection. In recent studies, the definition of sublobectomy includes the wedge resection and segmentectomy. Some of the studies analyzed the survival of patients after wedge resection or anatomic segmentectomy separately, whereas some studies studied only the survival after sublobectomy. We enrolled studies in which we could collect survival data after sublobectomy versus lobectomy to further analyze the outcomes of these results.

### Statistical analysis

The log (hazard ratio, ln (HR)) and its standard error (SE) were used as the outcome measure for data combination [14,15]. The SE was obtained as:

ln(upper 95% CI) - ln(lower 95% CI)/3:92

The ln (HR) and its SE were calculated from the reported data directly by HR and its 95% CI, or indirectly by reading and extracting from the Kaplan-Meier survival curve [5,9,12,16-24]. The heterogeneity of included studies was detected using the Cochran test. The publication bias was analyzed by Egger’s methods [25]. The evidence of asymmetry was based on *P* <1. Kaplan-Meier curves were read by Origin version 9 http://www.originlab.com. The data combining the test of heterogeneity was conducted and analyzed using Review Manager Version 5.1 http://www.cochrane.org/.

## Results

**Figure 1:**
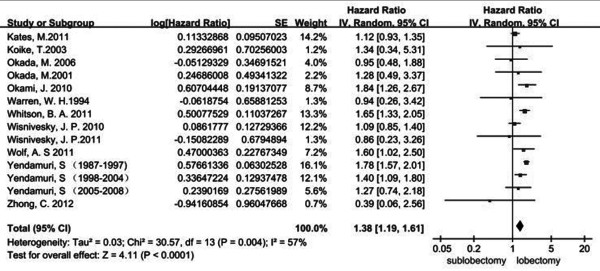
**Forest plot of HR for OS impact of operative approach (sublobectomy versus lobectomy) of stage IA NSCLC patients.** The combined HR displayed in this figure when compared with sublobectomy suggested that there was a significant benefit of lobectomy on OS of stage IA patients with tumors no larger than 2 cm, (HR 1.38; 95% CI, 1.19 to 1.61; *P* <0.0001) [5,9,12,16-24]. CI, confidence interval; df, degree of freedom; HR, hazard ratio, OS, overall survivalNSCLC,non-small cell lung cancer; SE, standard error

**Figure 2:**
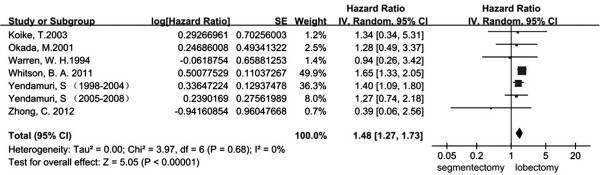
**Funnel plot of this analysis.** The crossed two lines in the figure represent the 95% CI. This figure presents the impact of operative approach (sublobectomy versus lobectomy) on OS of stage IA NSCLC patients with a tumor size of 2 cm or less.

**Figure 3:**
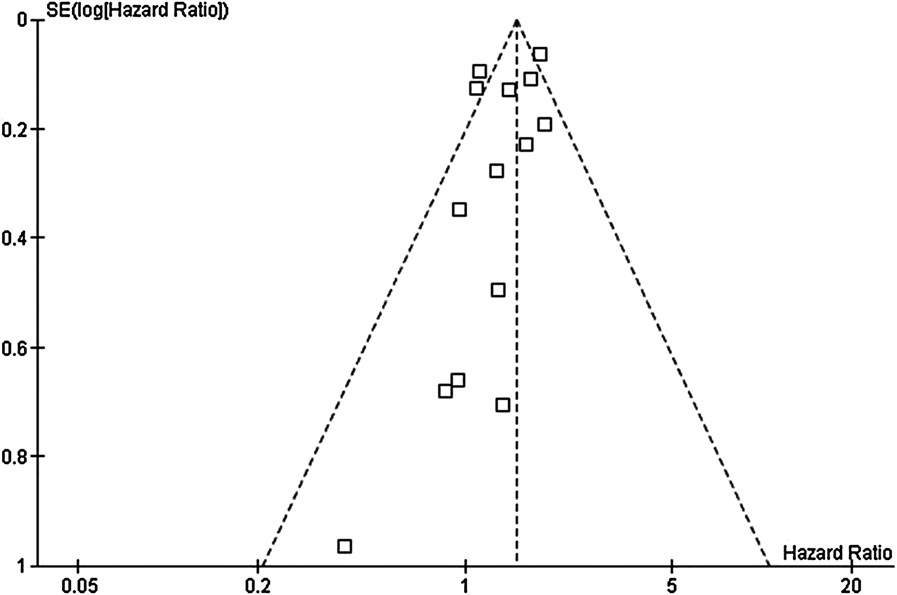
**Forest plot of HR for OS impact of operative approach (sublobectomy versus lobectomy) of stage IA NSCLC patients with data after 2000.** The combined HR displayed in this figure suggested there was no statistical significance between sublobectomy and lobectomy on OS (HR 1.38; 95% CI, 0.95 to 2.00; *P* = 0.09). CI, confidence interval; df, degree of freedom; HR, hazard ratio; OS, overall survival [16-18].

**Figure 4:**
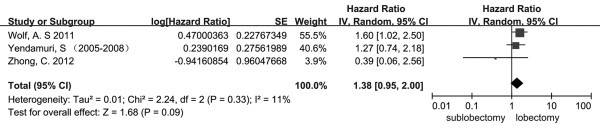
**Forest plot of HR for OS impact of operative approach (segmentectomy versus lobectomy) of stage IA NSCLC patients.** The combined HR displayed in this figure, compared with segmentectomy, suggest that there was a significant benefit of lobectomy on OS of stage IA patients with tumors no larger than 2 cm (HR 1.48; 95% CI, 1.27 to 1.73; *P* <0.00001)]. CI, confidence interval; df, degree of freedom; HR, hazard ratio; OS, overall survival [5,9,12,16,17,21].

The total number of studies obtained from the searches were 1342 from PubMed and Embase. According to the inclusion and exclusion criteria, there were 12 eligible studies published between 1994 and 2013 [5,9,12,16-24]. All of these studies were retrospective studies. The characteristics of all the included clinical trials are listed in Table 1. The size of the cohorts varied from 72 to 5626, with a total number of 18720 patients. In the analysis, the sublobectomy group was the experimental group and the lobectomy group was chosen as the reference. There were a total of 12 studies involved in the analysis that compared the impact of sublobectomy and lobectomy on OS of Stage IA (T1a) NSCLC patients. The combined HR of OS was 1.38 (95% CI, 1.19 to 1.61; *P* <0.0001). The sublobectomy group was inferior to patients treated with lobectomy. The Cochran tests for heterogeneity showed that tau^2^ = 0.03; chi^2^ = 30.57 df =13 (*P* = 0.004); I^2^ = 57%, which suggested significant inconsistency; and so we choose to use the random method instead random effects models (Figure 1). There was no significant publication bias detected at the section of analysis (Figure 2). Taking the age into consideration, we reasoned that the prognosis might be different in time periods. To minimize this deviation we excluded all data from before 2000 and made a future comparison. As a result, there were only three studies involved, and unfortunately, the outcome showed no statistical significance (HR 1.38; 95% CI, 0.95 to 2.00; *P* = 0.09) (Figure 3). In order to avoid the interaction between wedge resection and segmentectomy, we also made a further comparison between the segmentectomy group alone and the lobectomy group (Figure 4). The combined HR of OS was 1.48 (95% CI, 1.27 to 1.73; *P* <0.00001). The result is similar to that of the sublobectomy group. The Cochran tests for heterogeneity showed that tau^2^ = 0.00; chi^2^ = 3.97 df =6 (*P* =0.68); I^2^ = 0%.

**Table 1:** General characteristics of the enrolled studies

**Study**	**Stage**	**Tumor size**	**Comparison**	**Outcome**	**Sample size**	**Sublobectomy**	**Lobectomy**
Koike *et al.*[5]	Ia	≤2 cm	segmentectomy vs lobectomy	5-year survival	233	74	159
Okada *et al.*[9]	Ia	≤2 cm	sublobectomy vs lobectomy	5-year survival	567	305	262
Whitson *et al.*[21]	Ia	≤2 cm	segmentectomy vs lobectomy	5-year survival	5626	291	5335
Wolf *et al.*[18]	Ia	≤2 cm	sublobectomy vs lobectomy	5-year survival	238	154	84
Kates *et al.*[24]	Ia	≤1 cm	sublobectomy vs lobectomy	5-year survival	2064	688	1376
Okada *et al.*[23]	Ia	≤2 cm	segmentectomy vs lobectomy	5-year survival	209	70	139
Okami *et al.*[22]	Ia	≤2 cm	sublobectomy vs lobectomy	5-year survival	764	146	618
Warren and Faber [12]	Ia	≤2 cm	segmentectomy vs lobectomy	5-year survival	72	38	34
Wisnivesky *et al.*[20]	Ia	≤2 cm	sublobectomy vs lobectomy	5-year survival	1165	196	969
Wisnivesky *et al.*[19]	Ia	≤2 cm	sublobectomy vs lobectomy	5-year survival	249	47	202
Yendamuri *et al.* (1987-1997) [17]	Ia	≤2 cm	sublobectomy vs lobectomy	5-year survival	1961	469	1492
Yendamuri *et al.* (1998-2004) [17]	Ia	≤2 cm	segmentectomy vs lobectomy	5-year survival	2691	150	2541
Yendamuri *et al.* (2005-2008) [17]	Ia	≤2 cm	segmentectomy vs lobectomy	5-year survival	2761	162	2599
Zhong *et al.*[16]	Ia	≤2 cm	segmentectomy vs lobectomy	5-year survival	120	39	81

The sublobectomy group did not distinguish between segmentectomy and wedge resection. In the study of Yendamuri *et al.* in 2013 [17], three groups of data in different time periods were compared.

## Discussion

There are three original prospective randomized clinical trials about the comparison between sublobectomy and lobectomy. The earliest one is written by Ginsberg and Rubinstein in 1995 [6] and was excluded because the group division did not conform to our study. The other two trials were launched by The National Cancer Institute (NCI) (CALGB 140503) in 2008 [13] and The Japan Clinical Oncology Group with the West Japan Oncology Group (JCOG0802/WJOG4607L) in 2009 [26]; both comparing the survival of lobectomy and sublobectomy for small peripheral non-small-cell lung cancer (≤2 cm). Neither study has finished to date.

Before these studies publish their final results and conclusions, we used meta-analysis to combine current retrospective data. All the data included in our study were drawn from the retrospective studies. We found significant discrepancies among these studies for sample size, distribution of histological types, gender ratio, reasons for sublobectomy, and details of the operation. These differences might contribute to inter-study heterogeneity, therefore, the random model in RevMan was used for significant heterogeneities detection [27]. When considering the possible existence of publication bias among the enrolled studies, a test for publication bias was performed and no significant publication bias was detected after proper application of the above test.

The proper extent of pulmonary resection should achieve complete eradication of the malignancy and reduce the damage as much as possible. The operation project for patients with stage IA (T1a) NSCLC (tumor diameter ≤2 cm) remains controversial and has been under debate for several years.

We analyzed the impact of sublobectomy and lobectomy on the OS of NSCLC patients with stage IA (T1a) (tumors ≤2 cm). We found that the combined HR of OS was 1.38 (95% CI, 1.19 to 1.61; *P* <0.0001). The sublobectomy group was inferior to patients treated with the lobectomy. The segmentectomy group alone was similar to confirmed (HR 1.48, 95% CI, 1.27 to 1.73; *P* <0.00001). However, with recent data (data after 2000), we did not get the same results in the comparison between sublobectomy and lobectomy groups; there was no statistical significance (HR 1.38; 95% CI, 0.95 to 2.00; *P* = 0.09). Since there were only three studies in this comparison, we could not draw a definite conclusion, however, there could be a correlation between good prognosis and sublobectomy.

There are some limitations of this study:. (i) We could not collect and analyze data about the chemotherapy and radiotherapy information which might affect the survival of some patients. (ii) We consider the clinical stage more significant than the pathological one in choosing a surgical method, however, most of the retrospective studies did not describe clearly whether the stage was clinical or pathological; (iii) We think that the comparison in patients who can tolerate the lobectomy should be more powerful, however, most studies did not separate the lobectomy tolerance group and non-lobectomy tolerance group; (iv) Most studies did not take systematic or sampling lymphadenectomy into consideration, which could have an influence on the five-year survival rate; (v) Studies on the field of comparison between sublobectomy and lobectomy for stage IA (T1a) NSCLC have not taken the appearance on CT (pure solid, pure ground grass opacity (GGO), and part solid + GGO.) into consideration, which is quite important to the prognosis; and (vi) Retrospective studies could have some bias due to reasons which have been mentioned before.

## Conclusions

The current meta-analysis disclosed that sublobectomy (including wedge resection and segmentectomy) causes lower OS in stage IA (T1a) NSCLC patients. We suggest that lobectomy is the best optimal choice, which is in line with the recommendation from National Comprehensive Cancer Network (NCCN) Clinical Practice Guideline in Oncology for NSCLC version 2.2013. Considering the heterogeneity among studies and all data from retrospective studies, the results of the meta-analysis should be interpreted with caution and the data of studies previously mentioned should be supplemented with a further analysis in the coming future.

## Competing interests

The authors declare that they have no competing interests.

## Authors’ contributions

YL participated in the literature searching and performed the statistical analysis, and drafted the manuscript. CH participated in the literature searching. HL and YC participated in the design of the study. SL conceived of the study, and participated in its design and coordination and helped to draft the manuscript. All authors read and approved the final manuscript.
